# Mathematical modeling of tumor therapy with oncolytic viruses: Regimes with complete tumor elimination within the framework of deterministic models

**DOI:** 10.1186/1745-6150-1-6

**Published:** 2006-02-17

**Authors:** Artem S Novozhilov, Faina S Berezovskaya, Eugene V Koonin, Georgy P Karev

**Affiliations:** 1 National Center for Biotechnology Information, National Library of Medicine, National Institutes of Health, Bethesda, MD 20894, USA; 2 Department of Mathematics, Howard University, 2400 Sixth Str., Washington D.C., 20059, USA

## Abstract

**Background:**

Oncolytic viruses that specifically target tumor cells are promising anti-cancer therapeutic agents. The interaction between an oncolytic virus and tumor cells is amenable to mathematical modeling using adaptations of techniques employed previously for modeling other types of virus-cell interaction.

**Results:**

A complete parametric analysis of dynamic regimes of a conceptual model of anti-tumor virus therapy is presented. The role and limitations of mass-action kinetics are discussed. A functional response, which is a function of the ratio of uninfected to infected tumor cells, is proposed to describe the spread of the virus infection in the tumor. One of the main mathematical features of ratio-dependent models is that the origin is a complicated equilibrium point whose characteristics determine the main properties of the model. It is shown that, in a certain area of parameter values, the trajectories of the model form a family of homoclinics to the origin (so-called elliptic sector). Biologically, this means that both infected and uninfected tumor cells can be eliminated with time, and complete recovery is possible as a result of the virus therapy within the framework of deterministic models.

**Conclusion:**

Our model, in contrast to the previously published models of oncolytic virus-tumor interaction, exhibits all possible outcomes of oncolytic virus infection, i.e., no effect on the tumor, stabilization or reduction of the tumor load, and complete elimination of the tumor. The parameter values that result in tumor elimination, which is, obviously, the desired outcome, are compatible with some of the available experimental data.

**Reviewers:**

This article was reviewed by Mikhail Blagosklonny, David Krakauer, Erik Van Nimwegen, and Ned Wingreen.

## Open peer review

Reviewed by Mikhail Blagosklonny, David Krakauer, Erik Van Nimwegen, and Ned Wingreen. For the full reviews, please go to the Reviewers' comments section.

## Background

Mathematical modeling of virus-cell interaction has a long history. Grounded in the vast and diverse theoretical epidemiology field, these mathematical models serve as valuable tools to explain empirical data, predict possible outcomes of virus infection, and propose the optimal strategy of anti-virus therapy [1,2]. The unquestionable success of mathematical models of certain virus-host systems, in particular, HIV infection [3,4] provides for reasonable hope that substantial progress can be achieved in other areas of virology as well.

Equally extensive efforts have been dedicated over many years to mathematical modeling of cancer development. Stochastic models that take into account random mutations and cell proliferation proved to be useful in the context of epidemiology and statistical data [5] and for modeling cancer initiation and progression in terms of somatic evolution [6]. Deterministic models of tumor growth have proved valuable as well. Many of these have addressed avascular and vascular tumor growth taking advantage of methods borrowed from physics [7,8] but some use population ecology models to treat tumor as a dynamic society of interacting cells [9-11]. A variety of mathematical approaches contribute to modeling cancer progression from different standpoints and take stock of various factors affecting tumor growth ([12,13] and references therein).

Here we address a complex process that involves both virus-cell interaction and tumor growth, namely, the interaction of the so-called oncolytic viruses with tumors. Oncolytic viruses are viruses that specifically infect and kill cancer cells but not normal cells [14-17]. Many types of oncolytic viruses have been studied as candidate therapeutic agents including adenoviruses, herpesviruses, reoviruses, paramyxoviruses, retroviruses, and others [15,17]. Probably, the best-characterized oncolytic virus, that has drawn a lot of attention, is ONYX-015, an attenuated adenovirus that selectively infects tumor cells with a defect in the *p53 *gene [16]. This virus has been shown to possess significant antitumor activity and has proven relatively effective at reducing or eliminating tumors in clinical trials [18-20]. Thus, in studies of patients with squamous cell carcinoma of the head and neck the response rate was significantly higher (78%) in patients who received the combination of viral therapy and chemotherapy than in patients who were treated with chemotherapy alone (39%). Furthermore, a small number of patients who were treated with the oncolytic virus showed regression of metastases [15]. Although safety and efficacy remain substantial concerns, several other oncolytic viruses acting on different principles, including tumor-specific transcription of the viral genome, have been developed, and some of these viruses have entered or are about to enter clinical trials [15,21-23].

The oncolytic effect can result from at least three distinct modes of virus-host interaction [15,17]. The first mode involves repeated cycles of viral replication in the tumor cells leading to cell death and, consequently, to tumor reduction and, potentially, elimination. The second mode consists in low-level virus reproduction that, however, results in the production of a cytotoxic protein, which then causes cell damage. The third mode involves virus infection of cancer cells that induces antitumor immunity. Cancer cells possess weak antigens for host immune sensitization. Virus infection causes inflammation and lymphocyte penetration into the tumor, with the virus antigens eliciting increased sensitivity to tumor necrosis factor-mediated killing.

Although the indirect modes of virus cancer therapy based on production of cytotoxic proteins or antitumor immunity may be promising, direct lysis of tumor cells by an oncolytic virus is the current mainstream strategy. The interactions between the growing tumor and the replicating oncolytic virus are highly complex and nonlinear. Thus, to precisely define the conditions that are required for successful therapy by this approach, mathematical models are needed. Experiments on human tumor xenografts in nude mice have shown that the effect of oncolytic virus infection on tumors can range from no apparent effect, to reduction and stabilization of the tumor load (i.e., the overall size of a tumor), to elimination of the tumor [24]. Complete regression of tumors has been reported also in some patients treated with oncolytic viruses as part of clinical trials [25]. However, the simplest mathematical models describing a growing tumor infected with an oncolytic virus fail to incorporate all possible outcomes; in particular, these models do not allow tumor elimination [12,26]. Here, we present a conceptual model of tumor cells-virus interaction, which, depending on system parameter values, exhibits various behaviors including deterministic elimination of the cancer cells.

Several mathematical models that describe the evolution of tumors under viral injection were recently developed. Our model builds upon the model of Wodarz [12,26] but introduces several plausible modifications. Wodarz [12,26] presented a mathematical model that describes interaction between two types of tumor cells (the cells that are infected by the virus and the cells that are not infected but are susceptible to the virus so far as they have the cancer phenotype) and the immune system. Here, we consider only direct killing of tumor cell by an oncolytic virus and, accordingly, disregard the effects of the immune system. The resulting model has the general form



where *X*(*t*) and *Y*(*t*) are the sizes of uninfected and infected cell populations, respectively; *f*_*i*_(*X*, *Y*) *i *= 1, 2, are the per capita birth rates of uninfected and infected cells; and *g*(*X*, *Y*) is a function that describes the force of infection, i.e., the number of cells newly infected by the virus released by an infected cell per time unit. Note that there is no separate equation for free virions; it is assumed that virion abundance is proportional to infected cell abundance, which can be justified if free virus dynamics is fast compared to infected cell turnover [2]. The model also assumes that, upon division of infected cells, the virus is passed on to both daughter cells. This is certain for viruses that integrate into the tumor cell genome but this assumption should also be appropriate for non-integrating viruses because active virion production should result in a very high probability that the virus is transmitted to both daughter cells. The functions used by Wodarz [26] are



where *r*_1_, *r*_2_, *d*, *a*, *b*, *K *are non-negative parameters. The assumptions are that the tumor grows in a logistic fashion (possibly, with different rates of growth for the uninfected and infected tumor cells), and the incidence of infection is proportional to the product *XY*; the latter assumption is based on an analogy with chemical kinetics, namely, the law of mass action.

The main result of the analysis of model (1)-(2) consists in defining conditions required for maximum reduction of the tumor load. It has been suggested that "because we used deterministic model, the tumor can never go completely extinct but can be reduced to very low levels"; elimination of the tumor then might occur through stochastic effects which are not part of the model per se [26]. In contrast, here we show that a straightforward modification of model (1)-(2) can lead to dynamical regimes that describe deterministic elimination of the tumor cells.

Other mathematical models for tumor-virus dynamics are, mainly, spatially explicit models, described by systems of partial differential equations (PDE) (which is an obvious and necessary extension of ODE models inasmuch as most solid tumors have distinct spatial structure); the local dynamics, however, is usually modeled by systems of ODE that bear close resemblance to a basic model of virus dynamics [1]. Wu et al. modeled and compared the evolution of a tumor under different initial conditions [27]. Friedman and Tao (2003) presented a rigorous mathematical analysis of a somewhat different model [28]. The use of PDE for the virus spread is the main feature that distinguishes the model of Friedman and Tao [28] from the model of Wu et al. [27]. Recently, Wein et al. [29] incorporated immune response into their previous model [27]. In this new study, recent preclinical and clinical data were used to validate the model and estimate the values of several key parameters, and it has been concluded that oncolytic viruses should be designed for rapid intratumoral spread and immune avoidance, in addition to tumor-selectivity and safety [29]. In a more recent study, an analysis of an ODE system, which is a simplified approximation to the previously described PDE model and bears some similarities to the model of Wodarz, has been described [30]. Tao and Guo [31] recently extended the model of Wein et al. [29], proved global existence and uniqueness of solution for the new model, studied the dynamics of this novel cancer therapy, and explored an explicit threshold of the intensity of the immune response for controlling the tumor. Wodarz also developed an extension of his previous model to study advantages and disadvantages of replicating versus nonreplicating viruses for cancer therapy [32].

A distinct aspect of all these models is the description of the process of infection (or, if free virus dynamics is explicitly modeled, the virus-cell contact as well) using the law of mass action, which states that the rate of change of the uninfected cell population is proportional (if no demography effects are taken into account) to the product *XY *(where *X *and *Y *are as before, or *Y *stands for virus population if the latter is included into the model). Under mass-action kinetics and the assumption of infinitesimally short duration of contact and homogeneous mixing of the cell populations, the contact rate is proportional to the product *XY *of the respective densities. There are situations when mass action can be a good approximation; however, in many real-life cases, it is only acceptable when *X *~ *Y*, giving unrealistic rates when *X *>> *Y *or *X *<<*Y*. In particular, for large populations of cells, finite and often slow spread of the virus prevents it from infecting a large number of cells per infected cell per unit of time such that a more realistic approximation of the infection process is required. The assumption underlying mass action is that the contact rate is a linear function of density, *N *= *X *+ *Y*. At the other extreme, the contact rate might be independent of host density. Assuming that infected and uninfected hosts are randomly mixed, this would lead to transmission function of the form *bXY*/(*X *+ *Y*). This mode of transmission is often called 'frequency-dependent' transmission [33]. This transmission function makes sense when *X *>> *Y *or *X *<<*Y *because both extremely low and extremely high rates of transmission are excluded from the consideration. The mass action assumption, which goes back to the pioneering work of Kermak and McKendrick [34], has almost always been used for transmission in host-pathogen models, in some cases, non-critically. Other modes of transmission have been used [33], and, importantly, can yield quite diverse results. We emphasize that the mode of transmission determines probable responses of the system to control, so it is vital to identify the most appropriate approach to model transmission. In particular, mathematically, 'frequency-dependent' transmission, because of the non-analytical vector field at the origin, yields a qualitatively different outcome, compared to 'mass-action' transmission.

The model (1)-(2) is a version of the classical predator-prey model of a biological community first developed by Lotka [35] and Volterra [36] in 1925–1931; the term *bXY *describes the simplest correspondence between prey consumption and predator production similar to the law of mass action. A crucial element in models of biological communities in the form (1) is the functional response *g*(*X*, *Y*), i.e., the number of prey consumed per predator per time unit for the given numbers of prey *X *and predators *Y*. In the Volterra model and in model (1)-(2), this function is *bX*. Another well-known model has been developed by Holling [37] and has been widely applied in epidemiology [38]. Under this model, *g*(*X*, *Y*) = *bX*/(1 + *abX*), which takes into account the saturation effect. These two types of possible functional responses (and many others) do not depend on predator density, i.e., *g*(*X*, *Y*) = *g*(*X*), and, accordingly, have been named 'prey-dependent' by Arditi and Ginzburg [39]. In many cases, it is more realistic to assume that the functional response is ratio-dependent (*g*(*X*, *Y*) = *g*(*z*), where *z *= *X*/*Y *[39]). If we consider a Holling-type function *g*(*z*) = *bz*/(1 + *z*), then we again obtain



In (3), the meaning of *b *is the infection rate, i.e., the mean number of infections an infected cell can cause per unit of time. In the terminology of epidemic models, such a rate term would be said to reflect proportional mixing as opposed to homogeneous mixing [40].

The ratio-dependent models present a challenge with regard to their dynamics near the origin due to the fact that they are undefined at (0, 0). Berezovskaya et al. showed that, depending on parameter values, the origin can have its own basin of attraction in the phase space [41], which corresponds to the deterministic extinction of both species [40-43]. In the context of the interaction between oncolytic viruses and tumors, it is clear that the ratio-dependent models display original dynamic properties that could be directly relevant for the possibility of tumor eradication by virus therapy.

Here, we show that a plausible change of the dynamical system modeling the growth of two competing populations of cells, one of which is infected by a virus and the other one is not infected, can result in a remarkable change in the model dynamics. Moreover, the additional dynamical regimes, which do not emerge in the original model, might be particularly important with respect to the underlying biological problem, the oncolytic virus therapy for cancers.

## Results and discussion

### The model

We introduce our model through the incorporation of ratio-dependent process of infection (3) into the model of Wodarz [26] (system (1)-(2)). The model based on (1) and (3), which considers two types of cells growing in logistic fashion, has the following form:



where *X *is the size of the uninfected cell population; *Y *is the size of the infected cell population; *r*_1 _and *r*_2 _are the maximum per capita growth rates of uninfected and infected cells correspondingly; *K *is the carrying capacity, *b *is the transmission rate (this parameter also includes the replication rate of the virus); and *a *is the rate of infected cell killing by the virus (cytotoxicity). All the parameters of the model are supposed to be nonnegative. Model (4) is subject to initial conditions *X*(0) = *X*_0 _> 0 and *Y*(0) = *Y*_0 _> 0. We do not include a separate equation for the virus in model (4), and the initial conditions are given for uninfected and infected cells, assuming that, at the initial moment, the system already contains some cells infected by the virus; this should be taken into account when the results of analysis of (4) are compared with clinical or experimental data.

With an appropriate change of variables (*X*(*t*), *Y*(*t*), *t*) → (*x*(*τ*), *y*(*τ*), *τ*), the model can be simplified to a dimensionless form with a reduced number of independent parameters. This simplifies the mathematical analysis while preserving the essential properties of the model. There exist several formally equivalent dimensionless forms of the model with three parameters (which is, in the present model, the smallest possible number of parameters). The choice of the form and the specific combination of the new model parameters for the transition to the dimensionless form are defined by the biological goal of the study.

Here, our goal is to analyze system (4), mainly, with respect to its dependence on the cytotoxicity of viruses and on the force of infection, so the two parameters we are particularly interesting in are *b *and *a*, which represent the virus characteristics that, to some extent, can be controlled. We proceed to examine the qualitative behavior of model (4) as a function of parameters. The goal is to construct the phase-parameter portrait of system (4), i.e., to divide the parameter space into domains of qualitatively (topologically) different phase behaviors.

### Phase-parameter portrait of the initial model with mass-action kinetics of the infection process

For the sake of completeness and convenience of comparison, we present a full phase-parametric portrait of system (1)-(2) with *d *= 0, noting that the original paper of Wodarz [26] does not present such a full analysis. We let *d *= 0 in (2), to keep the number of independent parameters as small as possible, but still preserving their non-negativity. It can be shown that the system with explicit natural mortality (i.e., *d *≠ 0) can be put into form without this additional parameter, and both of the systems have topologically equivalent phase-parametric portraits.

Rescaling model (1)-(2) by letting

**Figure 1:**
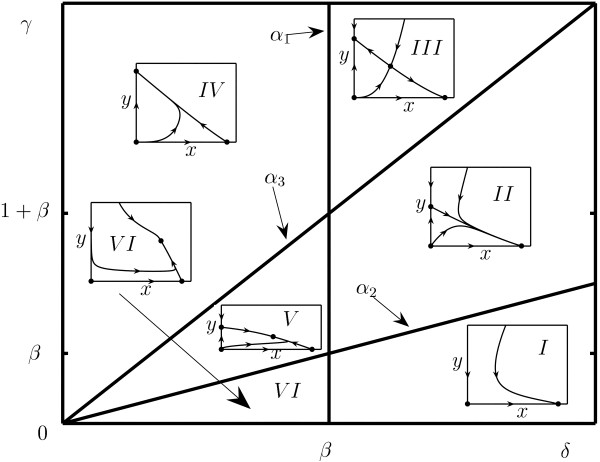
Phase-parameter portrait of system (5) given as a cut of the positive parameter space (*γ*, *β*, *δ*) for an arbitrary fixed value of *β *> 0. The boundaries between domains correspond to changes in behavior; the corresponding equations are listed in the text. Dots represent the model equilibria.

*x*(*τ*) = *X*(*t*)/*K*, *y*(*τ*) = *Y*(*t*)/*K*, *τ*= *r*_1_*t*

leads to the system



where *γ *= *r*_2_/*r*_1_, *β *= *bK*/*r*_1_, *δ *= *a*/*r*_1_. There exist six topologically different domains in the parametric space (*γ*, *β*, *δ*) (Fig. 1). The bifurcation boundaries of the domains in Fig. 1 are *α*_1 _= {(*δ*, *γ*, *β*) : *δ *= *β*}, *α*_2 _= {(*δ*, *γ*, *β*) : *γ *= *δ*}, and *α*_3 _= {(*δ*, *γ*, *β*) : *γ *= *δ *(*β *+ 1)/*β*}.

Fig. 1 shows that there are four different regions of parameter values in which the biological interpretation differs. These are: i) domains *I *and *II *where the asymptotic state of the system is characterized by absence of infected cells; ii) domain *III *where, depending on the initial conditions, the system can find itself either in the state where all the cells are infected or in the state where all the cells are uninfected; iii) domain *IV *where the final state of the system corresponds to the absence of uninfected cells (all cells are infected); and iv) domains *V *and *VI *where there is a globally stable inner equilibrium that corresponds to coexistence of both cell populations.

Possible biological implications of this analysis are discussed below; here, we only point out that all possible behaviors yielded by system (5) are also present in model (4) (together with additional dynamical regimes).

### Exponential growth of the cell populations

Prior to analyzing model (4) in full, it is worth examining its particular case when both cell populations grow unbounded under the exponential law, i.e., formally, 1/*K *= 0. The relevance of such a system is twofold. First, cancer cells are characterized by high proliferation ability, and the exponential growth of tumors is biologically realistic, at least, at early stages of tumorigenesis. Second, as shown below, the system with unlimited cell growth is the simplest mathematical model that possesses the property of having the elliptic sector (in biological terms, the simplest model that allows for elimination of both cell populations), and can serve as a building block to formulate and analyze more complex mathematical models.

The resulting system is



where *r*_1 _and *r*_2 _are per capita growth rates of uninfected and infected cells, respectively (since all the parameters of the model are supposed to be nonnegative, we keep parameter *a*). Choosing another time-scale *τ*= *r*_1_*t*, we obtain the system



where *x*(*τ*) = *X*(*t*), *y*(*τ*) = *Y*(*t*), *β *= *b*/*r*_1_, *γ *= *r*_2_/*r*_1_, and *δ *= *a*/*r*_1_. In the nondegenerate case *γ *- *δ *+ *β *- 1 ≠ 0, system (7) has the only equilibrium **O**(0, 0), and this equilibrium is singular.

The important mathematical peculiarity of system (7) is that the origin is a nonanalytical complicated equilibrium point. The structure of the neighborhood of point **O**(0, 0) in the first quadrant of the plane (*x*, *y*) and the asymptotes of trajectories for *x*, *y *→ 0 substantially depend on parameter values.

**Figure 2:**
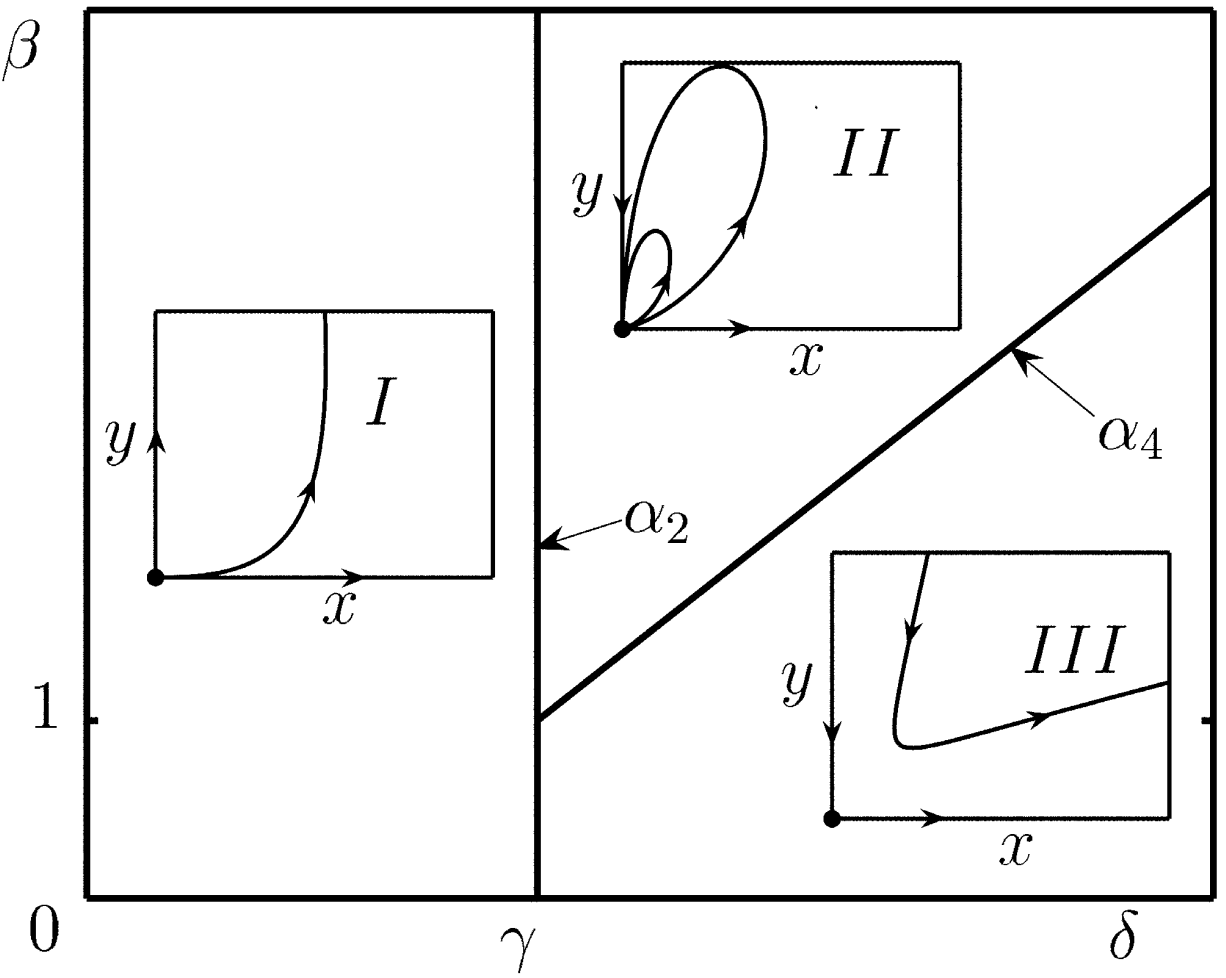
Phase-parameter portrait of system (7) given as a cut of the positive parameter space (*γ*, *β*, *δ*) for an arbitrary fixed value of *γ *> 0. The bifurcation boundaries are *α*_2 _= {(*δ*, *γ*) : *δ *= *γ*}, and *α*_4 _= {(*δ*, *γ*) : *β *= *δ *+ 1 - *γ*}

It is natural to continuously extend the determination of system (7) into the origin by changing the independent variable: *τ*→ (*x *+ *y*)*τ*. The structure of the point **O **as well as the asymptotes of trajectories with *x*, *y *→ 0 are shown in Fig. 2 and described in Lemma 1.

**Lemma 1. ***For different positive values of parameters δ*, *β*, *and γ, there exist three types of topologically different generic structures of the neighborhood of point ***O **(*and, accordingly, three topologically different phase portraits of system *(7)):

1) *a repelling-node sector *(*domain I in *Fig. 2) *for the parameter values δ *<*γ*. *The phase curves of the system which tends to ***O ***are of the form*

*y *= *Cx*^*γ *+ *β *- *δ *^(1 + *o*(1))     (8)

*if β *> *δ *+ 1 - *γ*,

*y *= *Cx*^(*γ *+ *δ*)/(1 - *β*) ^(1 + *o*(1))     (9)

*if β *<*δ *+ 1 - *γ*, *where C *≠ 0 *is an arbitrary constant*;

2) *an elliptic sector *(*domain II in *Fig. 2) *composed by trajectories tending to ***O ***as t *→ ∞ (*with asymptotic given by *(9)), *as well as with t *→ -∞ (*with asymptotic given by *(8)) *if δ *> *γ and β *> *δ *+ 1 - *γ *(*which necessarily yields β *> 1)

3) *a saddle sector *(*domain III in *Fig. 2) *for the parameter values δ *> *γ and β *<*δ *+ 1 - *γ*;

An elliptic sector is defined as a family of homoclinics that contains no inner equilibrium (see domain *II *in Fig. 2). Lemma 1 is proved in the Appendix (see Additional Files 1 and 2) by using the version of the blow-up method associated with the Newton diagram [44,45]. The phase-parameter portrait of (7) is given in coordinates (*δ*, *β*).

Thus, in spite of its apparent simplicity, system (7) demonstrates three different types of dynamic behavior, including the possibility to completely eliminate both cell populations in parameter domain *II *(Fig. 2). We use the results obtained for system (7) for analysis of a tumor cell-virus interaction model with the logistic growth law.

### Main properties of the system

If 1/*K *≠ 0 in (4) and there are no infected cells (*Y*_0 _= 0), then the tumor grows logistically, *X*(*t*) → *K *when *t *→ ∞; the applicability of this model to some tumors has been demonstrated experimentally [46]. Other mathematical forms, such as Gompertzian growth and power-law growth, also have been considered in the context of tumor growth and can be made to fit empirical data [47]. In general, there is no simple universal law to describe tumor growth [48]; we chose logistic growth because it is the simplest form whose predictions agree with the empirical data.

Re-scaling model (4) by letting

*x*(*τ*) = *X*(*t*)/*K*, *y*(*τ*) = *Y*(*t*)/*K*, *τ*= *r*_1_*t*

leads to the system



where *β *= *b*/*r*_1_, *γ *= *r*_2_/*r*_1_, and *δ *= *a*/*r*_1_. We proceed to study the qualitative behavior of model (10) as a function of parameters.

Let us consider the triangular region of 

Ω = {(*x*, *y*) ∈  : 0 ≤ *x *+ *y *≤ 1}.

By examining the direction of the vector field of system (10) on the boundary of Ω, it can be verified that Ω is positive invariant. Furthermore, if we assume that *x*(*τ*) + *y*(*τ*) > 1 with *x*(0) + *y*(0) > 1 is true for all *τ*> 0, then



Equation (11) leads to

**Lemma 2. ***Any trajectory of system *(10) *starting within **but outside *Ω *will enter *Ω *in a finite time*.

Hence, not only is Ω positive invariant, but it also is attractive to . Lemma 2 also states that any global stability in Ω is, essentially, the global stability in . We henceforth perform our mathematical analysis within the feasible domain Ω.

If we choose *D*(*x*, *y*) = 1/(*xy*) as a Dulac function, then



rules out the possibility of oscillations.

**Lemma 3. ***For any positive parameter values of δ*, *β*, *and γ, there is no closed trajectory to system *(10).

Since closed trajectories do not exist, equilibria play the key role in determining the dynamics of the model and will be analyzed below. There are four possible equilibria **O**(0, 0), **A**_1_(1, 0), **A**_2_(0,(*γ *- *δ*)/*γ*), and **A**_3 _= (*k*(*βγ *- *δ*), *k*(*δ *- *β*)) where

*k *= (*β *- 1 + *γ *- *δ*)/(*β *(*γ *- 1)^2^). Equilibrium **O**(0, 0) always exists. However, because neither *P*(*x*, *y*) nor *Q*(*x*, *y*) in (10) are analytic at this point, the linearization approach that is commonly employed to analyze the structure and the stability of this equilibrium fails. This issue received considerable attention in dynamical analysis of ecological models [39,40]. In spite of the fact that many epidemiological models with demography processes possess the same feature [38], only recently the existence and importance of this peculiarity were emphasized [40,41,43].

The standard approach to the analysis of this class of models involves the system



which is obtained from (10) with the change d*τ*→ (*x *+ *y*)d*τ*. Noting that the main part of (12), i.e., in this case, the terms of the second order, coincides with system (7), we can use the results described above for the latter system to obtain the structure of a positive neighborhood of the origin of system (10). Accordingly, the possible topologically nonequivalent cases are shown in Fig. 2.

The second equilibrium **A**_1_(1, 0) also always exists. The local stability of **A**_1_(1, 0) can be examined by the regular linearization approach. The Jacobian around **A**_1_(1, 0) is



hence the analysis of the corresponding linear system leads to proposition 1.

Proposition 1.

1) *If δ *> *β*, *equilibrium ***A**_1_(1, 0) *is a stable node whereas, if δ *<*β*, *it is a saddle;*

2) *The phase curves of the system which tend to ***A**_1_(1, 0) *are of the form*

*y *= *k*(*x *- 1)(1 + *o*(1)), *k *= -(*β *- *δ *+ 1)/(*β *+ 1).     (13)

*If ***A**_1_(1, 0) *is a saddle, then formula *(13) *and the two positive sections of the x-axis produced by ***A**_1_(1, 0)* determine its separatrices*.

Equilibrium **A**_2 _exists and belongs to Ω if *γ *> *δ*. The Jacobian around **A**_2 _is



hence the analysis of the corresponding linear system leads to proposition 2.

Proposition 2.

1) *If γ *> *δ*, *equilibrium ***A**_2 _*belongs to *Ω. *It is a saddle if δ *> *βγ and a stable node if δ *<*βγ *;

2) *The phase curves of the system which tend to ***A**_2 _*are of the form*

*y *= *kx*(1 + *o*(1)) + (*γ *- *δ*)/*γ*, *k *= -*γ *(*β *- *γ *+ *δ*)/(*γ *(*β *- *γ *+ *δ*) - *δ*).     (14)

*If ***A**_2_* is a saddle, them formula *(14) *and the two positive sections of the x-axis produced by ***A**_1_(1, 0) *determine its separatrices*.

The fourth potential equilibrium is **A**_3 _= (x^*^, y^*^) where



**A**_3 _belongs to Ω if one of the following two sets of conditions is satisfied:



Indeed, if (15) holds:



The local stability of **A**_3 _can be examined by noting that the determinant and trace of the Jacobian around **A**_3 _are of the form



If the first set of conditions in (15) is satisfied, then det**J**(**A**_3_) < 0, tr **J**(**A**_3_) < 0, and if the second set of conditions in (15) is satisfied, then det**J**(**A**_3_) > 0, tr **J**(**A**_3_) < 0; hence we have the following proposition.

**Proposition 3. ***If one of the two sets of conditions *(15) *holds, equilibrium ***A**_3 _*belongs to *Ω. *If β *> *δ*, *then ***A**_3 _*is an asymptotically stable topological node, and if β *<*δ*, *it is a saddle*

The global stability of **A**_3 _in case of *β *> *δ *follows from Proposition 3, Lemma 2, and Lemma 3.

**Proposition 4. ***The positive equilibrium ***A**_3 _*of system *(10) *is globally asymptotically stable in **if the second set of conditions *(15) *holds*.

### Phase-parameter portraits

**Figure 3:**
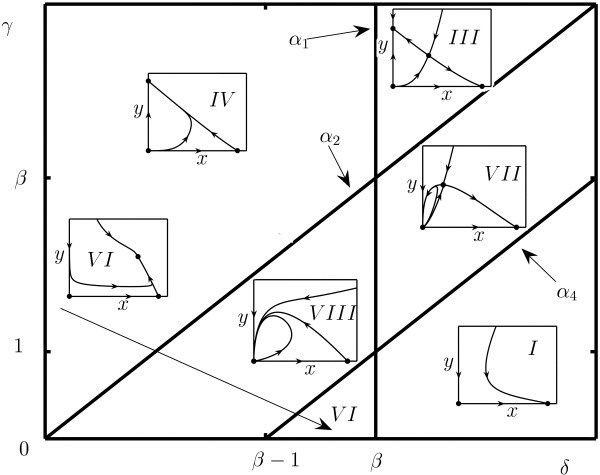
Phase-parameter portrait of system (10) given as a cut of the positive parameter space (*γ*, *β*, *δ*) for an arbitrary fixed value of *β *> 1. The dots represent the equilibria of the model. The cross-sections of the full three-dimensional parametric portrait are different for *β *< 1 (see text and Fig. 1) and *β *> 1 (the presented figure). The boundaries of the domains are *α*_1 _= {(*δ*, *β*, *γ*) : *δ *= *β*}, *α*_2 _= {(*δ*, *β*, *γ*) : *γ *= *δ*}, and *α*_4 _= {(*δ*, *β*, *γ*) : *γ *= *δ *+ 1 - *β*}.

In this section, we focus on the (*x*, *y*) -phase and (*δ*, *β*, *γ*) -parameter portrait of system (10). This phase-parameter portrait is obtained from the cuts on the (*δ*, *γ*) -plane generated by fixed values of *β*. Four lines partition the parameter space. Their equations are listed in the caption to Fig. 3 and in Theorem 1 below. The cut of the parameter portrait on the (*δ*, *γ*) -plane and the corresponding phase portraits critically depend on the value of *β *and are different for *β *< 1 and *β *> 1. The phase-parameter portrait in (*δ*, *γ*) -plane for the case *β *< 1 is almost exactly the same as for system (5) (Fig. 1). The minor difference comes from the equation for line *α*_3 _which, in case of system (10), is *α*_3 _= {(*δ*, *β*, *γ*) : *γ *= *δ*/*β*}, and, consequently, the intersection point of lines *α*_1 _and *α*_3 _has coordinates (*β*, 1) instead of (*β*, 1 + *β*). The phase-parameter portrait of system (10) in the case *β *> 1 is shown in Fig. 3.

The main mathematical result of the present work is formulated in Theorem 1.

**Theorem 1. ***The space of non-negative parameters *(*δ*, *β*, *γ*) *for system *(10) *is subdivided into 8 domains of topologically different phase portraits belonging to *Ω. *The cuts of the parameter space corresponding to fixed values of β are given in *Fig. 1*for β *< 1 *and in *Fig. 3 for *β *> 1. *The boundary surfaces between domains correspond to the following bifurcations for system *(10):

*α*_1 _= {(*δ*, *β*, *γ*) : *δ *- *β *= 0} *specifies the appearance/disappearance of equilibrium point ***A**_3 _*and the change of the topological type of point ***A**_1 _(*transcritical bifurcation*);

*α*_2 _= {(*δ*, *β*, *γ*) : *γ *- *δ *= 0} *specifies the change of the topological structure of equilibrium point ***O ***with the appearance/disappearance of point ***A**_2_;

*α*_3 _= {(*δ*, *β*, *γ*) : *γβ *- *δ *= 0} *for β *< 1 *specifies the appearance/disappearance of equilibrium point ***A**_3 _*and the change of the topological type of point ***A**_2_(*transcritical bifurcation*).

*α*_4 _= {(*δ*, *β*, *γ*) : *γ *- *δ - *1 + *β *= 0} *for β *> 1 *gives rise to the change of the topological structure of equilibrium point ***O ***with the appearance/disappearance of point ***A**_3_.

**Figure 4:**
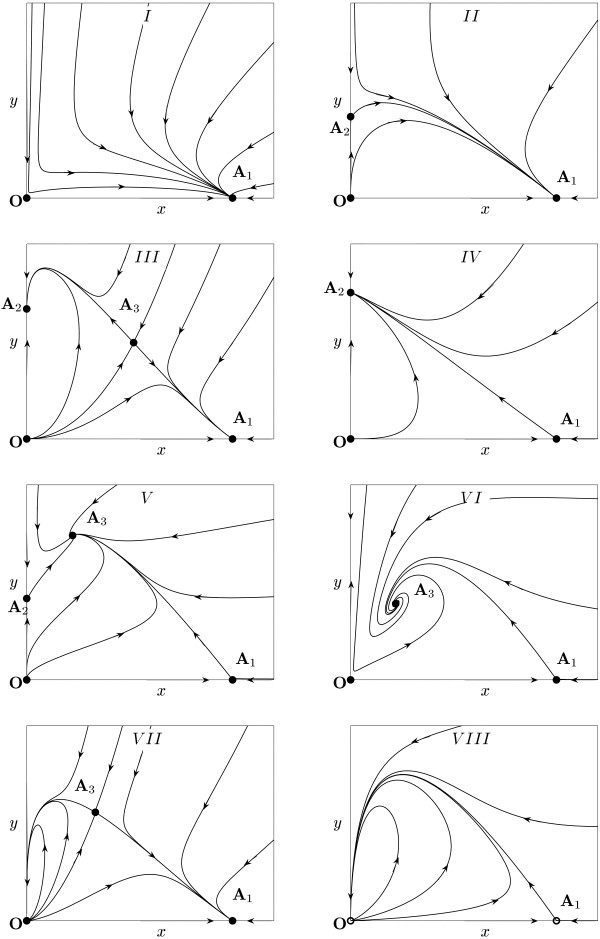
Topologically non-equivalent phase portraits of system (10) obtained by numerical simulation. The panels are numbered in accordance with the domains of the parameter space (Figs. 1 and 3). The parameter values used in numerical simulations are given in Table 1.

All boundary surfaces correspond to bifurcations of co-dimension one (the total number of "connections" between parameters) in system (10) [49]. Figures 1 and 3 represent the two-dimensional cross-sections of the parameter portrait of the system for *β *< 1 and *β *> 1, respectively. Our theoretical analysis is confirmed by numerical simulations as shown in Fig. 4 where the typical phase portraits of system (10) can be seen. The parameter values used in these simulations are listed in Table 1. Of particular interest is the occurrence of a family of homoclinics trajectories, which appear when the parameters are in domains *VII *and *VIII *in Figs. 3 and 4.

**Table 1:** Parameter values in phase portraits in Fig. 4

Parameter\domain	I	II	III	IV	V	VI	VII	VIII
*β*	1.5	0.5	1.5	1.5	0.5	1.5	1.5	1.5
*δ*	2	1	2	1	0.3	1	2	1
*γ*	1	1.8	2.5	2.5	0.5	0.3	1.8	0.7

Let us list the order of bifurcations that appear in system (10) for typical parameter values. First, assume that *β *> 1 and we start in domain *I *in Fig. 3 and move counter-clockwise such that each domain is visited. In domain *I*, there are two equilibria, **O **(saddle) and **A**_1 _stable node) (Fig. 4). On crossing *α*_4_, equilibrium **A**_3 _(saddle) breaks off **O**, which is accompanied by the appearance of an elliptic sector (Fig. 4, domain *VII *in Fig. 3). On line *α*_2_, the stable node **A**_2 _breaks off **O**, and the elliptic sector disappears (Fig. 4, domain *III *in Fig. 3). On line *α*_1_, a transcritical bifurcation occurs, with **A**_3 _coalescing with **A**_1_, and **A**_1 _changing its type to a saddle (Fig. 4, domain *IV *in Fig. 3). The next bifurcation occurs on *α*_2 _and is accompanied by the appearance of an elliptic sector; **A**_2 _coalesces with **O **(Fig. 4, domain *VIII *in Fig. 3). On line *α*_4_, the elliptic sector disappears, **A**_3 _(a stable equilibrium) breaks off **O **(Fig. 4, domain *VI *in Fig. 3). Finally, on *α*_1_, a transcritical bifurcation occurs where **A**_3 _coalesces with **A**_1_, and **A**_1 _changes its type to a stable node.

If *β *< 1 and we start from domain *I *in Fig. 1 and move counter-clockwise crossing every domain, the order of bifurcations is different. On *α*_2_, a saddle equilibrium **A**_2 _breaks off the origin, and **O **changes its type (domain *II *in Fig. 1). On *α*_3_, saddle equilibrium **A**_3 _breaks off from **A**_2_, and **A**_2 _changes its type to a stable node, which corresponds to a transcritical bifurcation (domain *III *in Fig. 1). On *α*_1_, **A**_3 _coalesces with **A**_1_. On *α*_3_, the globally stable equilibrium **A**_3 _breaks off **A**_2_, and **A**_2 _changes its type (domain *V *in Fig. 1). Finally, on *α*_2_, the saddle equilibrium **A**_2 _coalesces with the origin.

### Interpretation of the phase-parameter portraits

**Figure 5:**
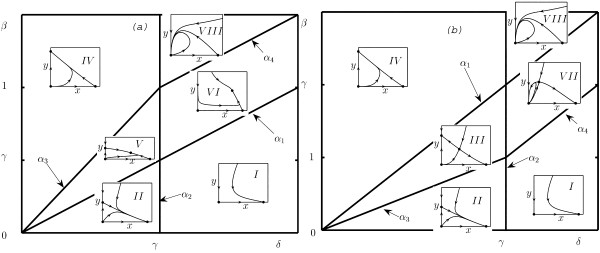
Phase-parameter portrait of system (10) given as a cut of the positive parameter space (*γ*, *β*, *δ*) for an arbitrary fixed value of 0 <*γ *< 1 (a) and 1 <*γ *(b). The boundaries of domains are listed in Theorem 1.

Various behaviors of model (10) in response to changes of the initial dimensional parameters can be easily interpreted in terms of the outcome of the oncolytic virus therapy. We use the bifurcation diagram shown in Figs. 1 and 3 and, for convenience, present the bifurcation diagram for a fixed arbitrary value of *γ *(Fig. 5) because the two parameters we are, mostly, interested in are *a *and *b *(*δ *and *β *in the dimensionless form). The bifurcation diagram (Figs. 1, 3, 5) demonstrates 8 types of system dynamics depending on the values of *δ*, *β *and *γ*. This reflects 6 biologically distinct types of behavior because domains *I *and *II *as well as domains *V *and *VI *are indistinguishable from the biological standpoint. The results of the present analysis permit us to completely describe the parametric domains where the tumor is eliminated, the domains in which virus infection stabilizes or reduces the tumor load, and the domains in which viral therapy fails to prevent tumor growth. Using the bifurcation diagram, it is easy to predict what happens when the system crosses the boundaries of the domains.

#### (i) Failure of viral therapy

Viral therapy fails if the eventual outcome of model dynamics is the globally asymptotically stable equilibrium **A**_1 _(domains *I *and *II *in Figs. 1, 3 and 5, see also Fig. 4). In terms of the dimensional parameters, the conditions for completely ineffective treatment are as follows:



It has to be emphasized that the result of viral treatment in this case does not depend on the initial conditions. For any initial values of *X*(0) > 0 and *Y*(0) > 0, the trajectories of system (4) as well as system (10) tend to the same asymptotical state that also would have been reached without virus administration. This suggests that, under given parameter values, even multiple, high-dose local administration of the virus to accessible tumors (a usual clinical practice) will be ineffective.

Note that, for this outcome, it is necessary (but not sufficient) to have *a *> *b*, i.e., the infection rate should be less than the death rate of infected cells caused by the virus' cytotoxicity. Under these conditions, the infected cells die without having time to infect other cells. In the framework of the present model, this situation, in part, could be a consequence of the assumption that virus dynamics is much faster than cell dynamics, which allows us not to model viral population dynamics explicitly. If virus dynamics is not fast compared to the turnover of the cells, explicit modeling of the virus population is required.

#### (ii) The bistable situation: potential success of viral therapy

For some parameter values, we can observe a situation when the final outcome of the therapy crucially depends on the initial conditions. This is the case for domains *III *and *VII *in the bifurcation diagram (Figs. 1, 3, 4 and 5). Depending on the initial conditions, the overall tumor cell population tends either to the maximum possible tumor load *X*(*t*) = *K *when *t *→ ∞), or to the equilibrium **A**_2 _in which all cells are infected but survive (domain *III*), or to the origin, i.e., complete elimination of the tumor cells (domain *VII*, the elliptic sector). The exact conditions for the bistable situation are as follows:

0 <*a *- *b *<*r*_2 _- *r*_1 _<*a *- *r*_1_

for the tumor elimination (domain *VII*); and



for stabilization of the tumor load at the equilibrium **A**_2 _(*Y*(*t*) = *K*(*r*_2 _- *a*)/*r*_2 _<*K*, *X*(*t*) = 0 when *t *→ ∞).

Several points are worth noting with regard to the bistable situation. First, the necessary condition to have a bistable situation is *r*_2 _> *r*_1_, i.e., the maximum per capita birth rate of infected cells should exceed the maximum per capita birth rate of uninfected cells which seems to be highly unlikely unless the virus triggers cell mechanisms that favor proliferation of infected cells over uninfected cells. Second, we again have the condition *a *> *b*, which indicates that viruses that kill cells with high efficiency but are poorly infective would have only a limited use in anti-tumor therapy (clearly, a biologically plausible conclusion).

Formally, domains *III *and *VII *differ significantly in the possible outcomes of virus therapy because, when parameters belong to domain *III*, it is only possible to stabilize the tumor size at the value *y *= (*γ *- *δ*)/*γ *= (*r*_2 _- *a*)/*r*_2_. In contrast, if the parameter values belong to domain *VII*, it is possible to eradicate the tumor (see Fig. 4), as indicated by the existence of the elliptic sector. Let us assume that there is a possibility to infect tumor cells instantaneously. Then, if the tumor size at the detection moment is *x*, the initial conditions for (10) are *x*(0) = *x *- *kx*, *y*(0) = *kx*, where 0 <*k *< 1. We can find a threshold value of *x *such that, if the tumor size at detection is larger than *x*, virus therapy becomes completely ineffective unless all tumor cells are infected (Fig. 5). The boundary in the phase space that divides the initial conditions into dangerous (we end up in *x *= 1) and favorable (we end up either in *x *= 0, *y *= (*r*_2 _- *a*)/*r*_2 _or in *x *= *y *= 0) is the separatrice of the saddle point **A**_3_.

#### 3.3. Stabilization or reduction of tumor load

Domains *IV*, *V*, *VI *(Figs. 1, 3, 4 and 5) are characterized by the presence of a globally stable equilibrium different from the maximal possible tumor load (*x *= 1 or *X *= *K*). This suggests that, by changing parameter values, the overall tumor load can be reduced to a finite minimal size. The analysis of this situation was one of the goals of Wodarz's work [26]. Note that, in this case, it is necessary to have *a *<*b*. The small total tumor load in the deterministic model corresponds to a real-life situation in which stochastic effects can eliminate tumor cells. Another possibility to eradicate the tumor in the deterministic setting is to lower the total tumor size to less that one cell.

**Figure 6:**
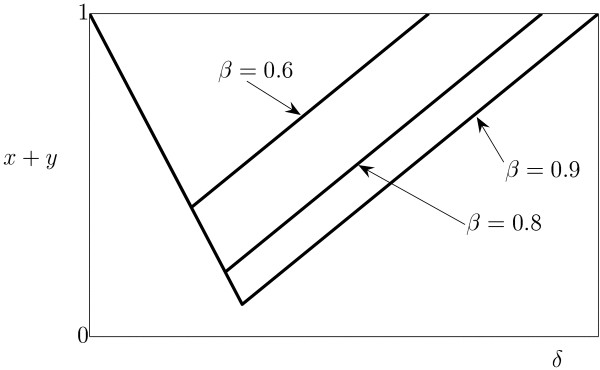
The overall tumor load *x*(∞) + *y*(∞) depending on viral cytotoxicity in the case of *δ *<*β *< 1. The value of *γ *is 0.3.

Let us consider the case of *β *< 1 (i.e., *b *<*r*_1 _in dimensional parameters). The overall tumor size *X*(*t*) + *Y*(*t*) is given by *K*(*r*_2 _- *a*)/*r*_2 _if all cells are infected (domain *IV*) and *K*(1 - (*b *- *a*)/(*r*_1 _- *r*_2_)) if the cell populations reach stable coexistence (domains *V *and *VI*). When *a *increases, first, we observe decrease of the tumor size, and then, as soon as *a *= *a*_*opt*_, the equilibrium tumor size starts to grow again (Fig. 6, the dimensionless parameter *δ *is used instead of the dimensional parameter *a*). The values of *a*_*opt *_correspond to the boundary between domains *IV *and *V*.

The optimal virus cytotoxicity is given by



and the minimal tumor size that can be reached is *K*(1 - *b*/*r*_1_). This means that, to reduce the tumor load significantly, we need to meet two conditions: *b *→ *r*_1 _and *a *→ *r*_2_. In dimensionless parameters, this means that we must have such values of parameters that the lines *α*_2 _and *α*_3 _coincide (Fig. 1) or at least are very close to one another. In other words, with fixed *β *< 1, it is impossible to choose values of *δ *and *γ *such that the tumor size becomes arbitrarily low, and the attainable tumor size might be large enough to prevent tumor elimination due to stochastic effects, which puts into question the generality of the conclusions of Wodarz [26] (see Fig. 6). Another important aspect of this situation is that, attempting to tune the parameters to maximally reduce the tumor load, we might find ourselves in highly unfavorable domains *I*, *II *or *III*.

#### (iv) Deterministic extinction of the tumor cell population

The most beneficial domain of parameter values in our analysis is domain *VIII *in Fig. 3. This domain corresponds to the total elimination of both cell populations (infected and uninfected) regardless of the initial conditions. This domain meets the most optimistic expectations for the use of replicating viruses for cancer therapy, i.e., that repeated cycles of infection, virus release, spread, and reinfection of tumor cells should eventually destroy the entire tumor. The conditions on parameter values for the system to be in this domain are



Note that the virus has to be highly infective in comparison with its cytotoxicity.

**Figure 7:**
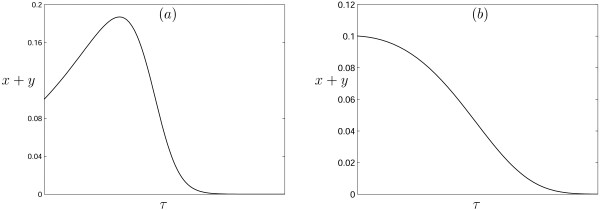
The tumor load versus time; parameter and initial condition values are: (a) *γ *= 0.7, *β *= 15, *δ *= 10, (b) *γ *= 0.7, *β *= 1005, *δ *= 1000. The initial conditions are *x*(0) = 0.1, *y*(0) = 0.0001.

Conditions (16) provide restrictions on parameter values to realize the regime of deterministic eradication of tumor cells; however, even if these conditions are met, on its way to extinction, the overall tumor size *X *+ *Y *can reach rather high values (which, with the parameters fixed, crucially depends on the initial conditions) (Fig. 7a). This indicates that we must not only identify the conditions that favor tumor elimination, but also develop the optimal strategy to infect initial tumor.

Let us assume that it is possible to instantly infect a particular part of the tumor. It is desirable to find the size of this part as a function of the model parameters such that the overall tumor size would monotonically decrease after infection. Since, for the maximum tumor load, *dx*/*dτ*+ *dy*/*dτ*= 0, from (10), we can write down the equation for the maximum tumor size: (*x *+ *γy*)(1 - (*x *+ *y*)) - *δy *= 0. Thus, if the tumor size at detection is , we obtain the expression



for the fraction of the cells that need to be instantly infected for the tumor load to decrease from the start of the viral therapy. Since *k*() is a monotonically decreasing function (*k*'() < 0 for any 0 < < 1), we can use the value

*k*(0) = 1/(1 + *δ *- *γ*) = 1/(1 + (*a *- *r*_2_)/*r*_1_)     (17)

to choose the parameters such as to minimize the percentage of the cells that have to be infected. From (16) and (17) it is clear that, ideally, one should have *b *> *a *>> *r*_2 _and *b *> *a *>> *r*_1_. As observed in experiments on human tumor xenografts in nude mice, when virus-infected tumor cells are mixed with uninfected tumor cells at the time of implantation, 1 cell in 1000 infected with Ad337 was sufficient to prevent tumor establishment and eventually eliminate all tumor cells [24]. Our results show that this situation readily merges within the framework of the model described here (Fig. 7b).

## Conclusion

In this work, we present the complete qualitative analysis of the deterministic model of the interaction of an oncolytic virus with tumor cells [system (10)] along with the auxiliary system (7). System (7) is the simplest mathematical model that includes the elliptic sector and can be used as a building block for other models.

We showed that:

i) all behaviors from previously described by Wodarz [26] are present in (10) along with additional dynamical regimes;

ii) one of the additional dynamical regimes discovered here (domain *VIII*) is of particular interest from the biological and therapeutic standpoints because it demonstrates the possibility of complete eradication of the tumor by virus therapy;

iii) our model, in contrast to original the model of Wodarz [26], exhibits all possible outcomes of oncolytic virus infection, i.e., no effect on the tumor, stabilization or reduction of the tumor load, and complete elimination of the tumor;

iv) the conditions on parameters were identified such that the initial infection results in immediate decrease of the tumor load and eventual elimination, and, for the given parameter values, the fraction of the tumor cells that has to be infected was found.

Although the available data are insufficient to rigorously validate the present model, it is notable that the fraction of the cells that have to be infected in order to achieve the most beneficial results within the model's framework is comparable to the values reported in experimental studies on tumor implantation in nude mice [24]. Clearly, the model described here is oversimplified, at least, in that it ignores virus population dynamics and immune system response; inclusion of parameters that characterize these and other factors may lead to more realistic models of virus-tumor interaction.

## Authors' contributions

ASN, FSB, and GPK developed the mathematical models; EVK incepted the study and provided the biological interpretations; ASN and FSB wrote the initial draft of the paper and EVK produced the final version; all authors edited and approved the manuscript.

## Reviewers' comments

Reviewer's report 1

Mikhail Blagosklonny, Cancer Center, Ordway Research Institute, Albany, NY, USA

This reviewer provided no comments for publication.

Reviewer's report 2

David Krakauer, Santa Fe Institute, Santa Fe, NM, USA

The appreciation of cancer as an evolutionary and ecological dynamic within the context of a multicellular organism has allowed many ideas developed in evolution and ecology to migrate across into the study of disease. Best known among these ideas are studies of virus dynamics and immune regulation.

Cancer cells share many dynamic properties with viruses including a rapid rate of proliferation, a high rate of mutation and an elicitation of immune responses. Both deterministic and stochastic evo-eco models have been developed to describe the time course of cancer emergence and cancer cell proliferation.

A number of researchers have thought of coupling these two dynamics (virus and cancer) in an effort to use some of the pathological properties of cancer to mitigate the pathological properties of cancer cells. In particular, encourage viruses to infect cancer cells (oncolytic virus) in order to promote cancer cell clearance through several routes including cytotoxicity and immune activation.

This paper represents an extension of existing work by Wodarz and colleagues to include an alternative non-linear functional response (Holling-type) for infection rate and a more exhaustive analysis of the parametric dependencies of the steady states. The parameters of focal interest are the coefficient of infected cancer cell death and the coefficient of infection.

The analysis is thorough and the extensions are interesting. The appearance of several different outcomes according to parameter values is valuable as it makes clear the potential complexity of behavior in a very simple dynamical system describing disease. The value of such a paper is to use quantitative models to make a qualitative point, that the outcome of biologically based therapies can be diverse and require model-based thinking as a guide to experiment.

The paper does however make a number of very strong assumptions, which limit its use as a therapeutic tool and make comparisons to extant data sets somewhat difficult. I list these assumptions below. I think it worth pointing these out, as they are significant issues for any comparable research into this area.

1. Cancer is an inherently stochastic process dependent on random mutation accumulation and a very noisy (sampling noise) process of cell proliferation when cancer cell numbers are low. Both mutation and extinction near the zero cell boundary condition are left out of this model. This has been treated extensively in some recent cancer models that the authors should probably read and cite. I am thinking of the early papers by Moolgavkar and the more recent models by Murray et al, Speer et al and Nowak et al.

**Author response: ***Cancer is certainly a stochastic process affected by random mutations and cell proliferation. We added to the text some recent references on stochastic modeling of tumorigenesis. However, we do believe that deterministic models are of significant importance in our understanding of cancer. A number of references to deterministic models were also added. Our model does not contain mutation process, but our basic goal was to construct a mathematical model, as simple as possible, that could produce all the regimes which were observed in experiments. Also, it has to be kept in mind that what is being modeled here is the interaction between a tumorolytic virus and the tumor, rather than the process of tumor growth per se, so not all stochastic aspects of the latter are necessarily relevant*.

2. Cancer tissue exists within a larger context of healthy tissue and density dependent growth does not only depend on the density of cancer cells as is assumed in this model but the density of healthy cells. While the model treats cell death in the usual phenomenological framework of logistic growth, some consideration of the mechanisms at work such as limitations in blood supply would be valuable, including research into the effects of angiotensin.

**Author response: ***Certainly, healthy tissues are important for tumor growth. Indirectly, this is implied in the logistic model of tumor growth, which is adopted here, as the growth may be limited by the effects of the healthy tissues. We do not believe that including further details into a simple model considered here would be productive*.

3. The separation of time scales which allows the free virion to be treated algebraically is perhaps less acceptable in the case of cancer than in virus infection of normal cells. The reason for this is that the cancer cell can have an elevated rate of proliferation, which would render this assumption inappropriate.

**Author response: ***This definitely might be the case, but we think that our description can still be applied to many real situations. Besides, incorporating virus population dynamics explicitly would yield a considerably more complex system of differential equations, which could hamper the comprehensive mathematical analysis of nonanalytical vector field. We agree that this suggestion is a reasonable next step to improve the model*.

4. The assumption of perfect viral specificity is unlikely to be possible and it is therefore important to quantify the cost of tumor therapy in terms of the reduction in the healthy population of cells in relation to the reduction in cancerous cells.

**Author response: ***Yes, this is a simplification, we cannot argue that, but it is one that is necessary to construct a model that can be not only written down but also analyzed in detail*.

5. Elimination might not be the desired outcome of therapy but control. We have no real way of knowing whether small micro-metastatic tumors have serious implications on individual morbidity and so alternative treatments that focus on protracted control could prove very interesting.

**Author response: ***Actually, it seems like elimination of a tumor always is the most desirable outcome. It is quite another matter that, this not being practical in a particular situation, control might be effectively as good. What we show here, is that, under certain regimes of virus-tumor interaction, complete, deterministic elimination of the tumor cell population is a possibility*.

6. Finally and perhaps most difficult for a model is the whole question of a quantitative statement of "disease". We can model individual cell populations but we do not really understand the relationship of the cell level to the individual clinical symptoms. How might we go about relating these two spatial scales within a common theoretical framework? I think this is an important question that deserves more attention from the research community and certainly goes beyond the realistic ambition of the present study.

**Author response: ***We just must agree and confirm that, perhaps, sadly, this is beyond our ambitions in the foreseeable future*.

Reviewer's report 3

Erik van Nimwegen, Division of Bioinformatics, Biozentrum, University of Basel, Basel, Switzerland

Novozhilov et al. study the phase portrait of a simple mathematical model for the dynamics of an oncolytic virus and tumor cells. This model is a particular example from the class of Lotka-Volterra like models and is an extension of a model proposed in the same context by Wodarz. The main feature of interest is that, as a function of the small set of parameters, the model exhibits qualitative different kinds of dynamical behavior such as coexistence of tumor and infection, disappearance of infection, disappearance of all tumor cells, etcetera.

First, a remark as to the novelty of the research here proposed. I do not know to the literature well enough to be able to point to a specific reference in which the model (10) has been studied but I would be quite surprised if this model, and its phase-portrait, had not already been described in the literature on Lotka-Volterra type models. I therefore expect that the main addition to the literature is the application of this model to the oncolytic virus system, and a discussion of its phase portrait in this context.

**Author response: ***This model is not exactly in the class of Lotka-Volterra models. The main difference (considering this model as a model of interaction of two species) is that the functional response is ratio-dependent, and this was the main topic of the work of Arditi and coauthors who emphasized the qualitative differences between these models and the classical Volterra models*.

This brings me to the main criticism of the manuscript. The presentation is rather mathematical throughout and will be completely inaccessible to most biomedical researchers. Even I had some difficulties with some of the terminology. For example I had to look up what an "elliptic sector" is (the definition in the paper didn't help me very much) and I also had to look up the Bendixson-Dulac theorem to understand how Lemma 3 follows. I bring this up mainly because I don't believe it is necessary to make the manuscript so technical. I think the main interest of the results lies in discussing the meaning of the phase-portrait for tumor therapy. The discussion does a reasonable job of this but I think it could be made even clearer to the point that the main conclusions would be easily accessible for biomedical researchers.

**Author response: ***The existence of the elliptic sector is the crucial aspect of the model presented in this paper. This mathematical notion is not just a technical detail but rather has important biological implications, i.e., in this particular case, the possibility of deterministic elimination of the tumor. Therefore we felt that it was necessary to include the required proofs*.

Some weight is put in the discussion of the mathematics on the fact that "The important mathematical peculiarity of system (7) is that the origin is a nonanalytical complicated equilibrium point". It seems to me that such mathematical subtleties could be easily avoided by expressing the equations in terms of the total number of tumor cells *n *= *x *+ *y *and the fraction *ρ *= *y*/(*x *+ *y*) of tumor cells that is infected. In terms of these variables the equations (7) become:



and



First, these equations are now analytic everywhere in the strip *n *≥ 0, 0 ≤ *ρ *≤ 1. Furthermore, the second equation does not depend on *n *and can be directly integrated to obtain explicit analytic expressions for *n*(*t*) and *ρ*(*t*). Even more simply, it is immediately apparent from the second equation that the sign of *β *- *δ *+ *γ *- 1 determines if the system will flow to *ρ *→ 1 as *t *→ ∞ or to *ρ *→ 0. And once we know what values *ρ *will limit to, we can fill its value in the first equation to see that the tumor will only disappear if *β *- *δ *+ *γ *- 1 > 0 and *δ *> *γ*. Obviously the authors reach the same conclusions but I think the derivation just sketched is much simpler and more easily accessible to the readership of Biology Direct than the rather technical derivation that the authors present.

Similar remarks apply to the system (10). If one again transforms to *n *and *ρ *one obtains



and



Here one cannot explicitly solve for *n*(*t*) and (*t*) in closed form but the system is analytic everywhere in the strip and the fixed points, their Jacobians, and the eigenvalues of these Jacobians can be easily determined. Again one would of course reach the same conclusions as the authors^1 ^but I think the mathematics is again substantially simplified in this coordinate system.

**Author response: ***The models (1)-(2) and (3)-(4) proposed by the reviewer are indeed equivalent to our models (7) and (10), respectively, only if the total population size n *= *x *+ *y is constant or, at least, is bounded from below, i.e., n *≥ *Const *> 0. *Otherwise, however, the systems are not equivalent and exhibit qualitatively different behaviors*.

On page 16 it is said "Noting that the main part of (12) coincides with system (7), we can use the results described above for the latter system to obtain the structure of a positive neighborhood of the origin of system (10)".

I presume that the expression "main part of (12)" is a technical term and that the authors are implicitly using a theorem here (that I am unfamiliar with) that proves that the topology of the set of trajectories close to the origin is the same in system (10) and system (7). Some more explanation would be helpful.

**Author response: ***The requisite explanations have been incorporated in the revision*.

Reviewer's report 4

Ned S. Wingreen, Department of Molecular Biology, Princeton University, Princeton, NJ, USA

The authors address the question of the possible outcomes of tumor therapy with oncolytic viruses within a simple but appropriate mathematical framework. The model allows for two populations of cells: uninfected tumor cells and infected tumor cells. The concentration of viral particles is not explicitly followed, and the tumor is assumed to be homogeneous in space, i.e. no spatial dependence of cell concentration is considered. As such, the system consists of two coupled, deterministic differential equations allowing for cell reproduction and death, and cell infection. Previous related work by Wodarz (Refs. 12, 26, and 32) assumed an infection rate proportional to the product of infected and uninfected cell concentrations (a la the law of mass action). Within this assumption, the equations never lead to elimination of the tumor. The main result obtained by Novozhilov et al. is that a plausible change in the form of the infection rate can lead, in some range of parameters, to additional outcomes in which all tumor cells, or either infected or uninfected cells can be completely eliminated. This is an important conclusion in terms of the medical implications of oncolytic viral therapy.

The paper is very clearly written, and, while rather technical, the mathematical analysis of the coupled equations for cell concentrations is enriched by some nice qualitative discussion of what the assumptions and results mean. Mathematically, the use of a ratio-dependent infection rate (~ XY/(X+Y), where X and Y are the uninfected and infected cell populations) leads to interesting behavior in the limit of low cell concentrations, and the authors do a good job of deriving and explaining this behavior.

Overall, I found the paper both interesting and informative. The careful study of a simple model is very instructive, and is likely to be helpful in guiding future work on oncolytic viral therapy.

A few minor comments on the manuscript:

1. It would have been helpful to have a more detailed review of the experimental status of oncolytic viral therapy in the introduction. The sentences leading up to citations to Ref. 18–20 give no quantitative sense of how effective this therapy is compared to any control experiments.

**Author response: ***We added one piece of quantitative information. We were reluctant to get deeper into that because the reliability of quantitative assessment of the comparative effects of different therapeutic strategies is a matter of concern*.

2. The discussion of the use of a ratio-dependent infection rate is rather terse considering how important this is to the manuscript. It is clear that in the limits X << Y and Y << X, the form used is sensible, but this is never discussed clearly. Similarly, it is never explained why, mathematically, the ratio-dependent and alternative form of the infection rate lead to qualitatively different outcomes. Again, this seems central to the paper, and deserves some more attention.

**Author response: ***In the revision, the reasons for adopting a ratio-dependent infection rate in our model are discussed in greater detail*.

## Note

^1^Here I am assuming the authors' derivations are all correct. I have not explicitly reproduced them all but all calculations that I did all confirmed the authors' results so I am confident that the derivations are indeed all correct.

## Supplementary Material

Additional File 1 Mathematical Appendix Click here for file

Additional File 2 Figure for the Mathematical Appendix Click here for file
