# Supplementary material for: Mathematical modeling of tumor therapy with oncolytic viruses: Regimes with complete tumor elimination within the framework of deterministic models
Source: Biol Direct. 2006 Feb 17;1:6. doi: 10.1186/1745-6150-1-6 (PMC1403749; doi:10.1186/1745-6150-1-6)
Supplement: Additional File 1 — Mathematical Appendix [file 1745-6150-1-6-S1.doc]

**Appendix**

*Proof of Lemma 1.* System (7) is analytical in all points of the plane $(x,y)$ except for the origin. The positioning of its phase trajectories in the first quadrant is identical to those of the polynomial system

$\begin{matrix} \frac{\text{d}x}{\text{d}\tau}=x(x+y)-\beta xy, \\ \frac{\text{d}y}{\text{d}\tau}=\gamma y(x+y)+\beta xy-\delta y(x+y) \end{matrix}$ (A.1)

obtained from (7) by a change of the independent variable $\tau\to(x+y)\tau$. System (A.1) has a complicated equilibrium point at the origin (because both eigenvalues are equal to zero) which is investigated below using the methods previously developed [1, 2].

The first step consists in a change of variables in system (A.1) (see also [3])

$$x=x,\text{ }u=y/x,\text{ }x\text{ d}\tau=\text{d}s$$

that transforms in a non-degenerate way the first quadrant of the $(x,y)$-plane, except $x=0$*,*  into the first quadrant of the $(x,u)$-plane and blows-up the point $\mathbf{O}$ into the $u$-axis. The resulting system is

$$\begin{matrix} \frac{\text{d}x}{\text{d}s}=x\left(1+(1-\beta)u\right)\text{ }, \\ \frac{\text{d}u}{\text{d}s}=u\left(\gamma-1+\beta-\delta+(\gamma-1+\beta-\delta)u\right)\text{ }, \end{matrix}$$

which has only one equilibrium point on the $u$-axes: $\mathbf{O}_{1}(0,0)$ (we are only interested in nonnegative equilibria). The eigenvalues of this point are $\lambda_{1}(\mathbf{O}_{1})=1$, and $\lambda_{2}(\mathbf{O}_{1})=\gamma-1+\beta-\delta$, hence this point is non-degenerate if $\gamma\neq\delta+1-\beta$. If $\gamma>\delta+1-\beta$ then $\mathbf{O}_{1}(0,0)$ is an unstable node, while it is a saddle if $\gamma<\delta+1-\beta$ (see also Fig. 2 and A1). In the case of unstable node, $\mathbf{O}_{1}(0,0)$ is the source of a family of trajectories with asymptotes

$$u=Cx^{\gamma+\beta-\delta-1}(1+o(1))$$

where $C\neq0$ is an arbitrary constant. In coordinates $(x,y)$ this family is transformed into family (8).

We now repeat the blow-up procedure to study the behavior of the system close to the $y$-axes:

$y=y,\text{ }v=x/y,\text{ }y\text{ d}\tau=\text{d}s$.

This transformation is non-degenerate for all values of $x,y$ except $y=0$ and the point $\mathbf{O}(0,0)$ blows-up into the $v$-axes. In variables $(v,y)$ we obtain the system

$\begin{matrix} \frac{\text{d}v}{\text{d}s}=v\left(1-\beta-\gamma+\delta+(1-\beta-\gamma+\delta)v\right)\text{ }, \\ \frac{\text{d}y}{\text{d}s}=y\left(\gamma-\delta-(\beta+\gamma-\delta)v\right)\text{ }. \end{matrix}$ (A.2)

This system has equilibrium $\mathbf{O}_{2}(0,0)$ on the $v$-axes with eigenvalues $\lambda_{1}(\mathbf{O}_{2})=\delta+1-\beta-\gamma$ and $\lambda_{2}(\mathbf{O}_{2})=\gamma-\delta$. Depending on the parameter values the following cases are realized in system (A.2) in the first quadrant of the $(v,y)$ plane (Fig. A1):

(i) $\mathbf{O}_{2}(0,0)$ is a saddle with $v$-axes stable manifold if $\gamma>\delta$ and $\beta>\delta+1-\gamma$;

(ii) $\mathbf{O}_{2}(0,0)$ is a unstable node if $\beta<\delta+1-\gamma$ and $\gamma>\delta$;

(iii) $\mathbf{O}_{2}(0,0)$ is a saddle with $y$-axes stable manifold if $\gamma<\delta$ and $\beta<\delta+1-\gamma$;

(iv) $\mathbf{O}_{2}(0,0)$ is a stable node if $\gamma<\delta$ and $\beta>\delta+1-\gamma$;

In cases (ii) and (iv) equilibrium $\mathbf{O}_{2}(0,0)$ is the source of the family of trajectories

$v=Cy^{(\delta+1-\beta-\gamma)/(\gamma-\delta)}(1+o(1))$.

Returning to original coordinates we obtain family (9).

Assembling together the obtained results and returning to the initial variables $(x,y)$ as shown in Fig. A1, we obtain different topological structures of the complicated point $\mathbf{O}(0,0)$ in the first quadrant of the plane $(x,y)$ depending on the system parameters.

Note that the phase portraits in the neighborhood of $\mathbf{O}$ in Figs. A1a and A1d are topologically equivalent and differ only in the asymptotes of characteristic trajectories. Therefore, there are only three topologically different structures in the plane $(x,y)$ in non-degenerate cases; they are presented in Fig. A1.

Proof of Lemma 1 is now complete.

Figure legends

**Fig. A1.** Phase portraits of the positive neighborhood of $\mathbf{O}$, which correspond to different domains in Fig. 2, together with phase portraits of auxiliary systems (see text), invoked for analysis of the singular equilibrium $\mathbf{O}$. The parameter values used in numerical simulation are given in Table A1

**Table A1.** Parameter values in phase portraits of Fig. A1

| Parameters: | (a) | (b) | (c) | (d) |
| --- | --- | --- | --- | --- |
|  | 1.5 | 1.5 | 1.5 | 0.5 |
| $\delta$ | 1 | 1 | 1 | 1 |
| $\gamma$ | 2 | 0.7 | 0.3 | 1.3 |

**References**

1. Berezovskaya FS: **About asymptotics of trajectories of a system of two differential equations**. *Report deposited in the All-Union Information Center (USSR), No 3447-76, 17 p (in Russian)* 1976.

2. Berezovskaya FS: **The main topological part of plane vector fields with fixed Newton diagram**. In: *Proceedings on Singularity Theory.* Edited by Le DT, Saito K, Teissier B. Singapore et al: Word Scientific; 1995: 55-73.

3. Jost C, Arditi R: **Identifying predator-prey processes from time-series**. *Theor Popul Biol* 2000, **57**(4):325-337.
